# Supplementary material for: Otilonium bromide boosts antimicrobial activities of colistin against Gram-negative pathogens and their persisters
Source: Commun Biol. 2022 Jun 21;5:613. doi: 10.1038/s42003-022-03561-z (PMC9213495; doi:10.1038/s42003-022-03561-z)
Supplement: Supplementary file 2 — Supplementary Information [file 42003_2022_3561_MOESM2_ESM.pdf]

Supplementary Materials

Otilonium bromide boosts antimicrobial activities of colistin against Gram-negative pathogens and their persisters

Chen Xu[1], Chenyu Liu[1], Kaichao Chen[1], Ping Zeng[2], Edward Wai Chi Chan[1,2], Sheng Chen[1*]

Running title: Otilonium bromide as colistin adjuvant

[1]Department of Infectious Diseases and Public Health, Jockey Club College of Veterinary Medicine and Life Sciences, City University of Hong Kong, Kowloon, Hong Kong;

[2]State Key Lab of Chemical Biology and Drug Discovery, Department of Applied Biology and Chemical Technology, The Hong Kong Polytechnic University, Hung Hom, Kowloon, Hong Kong;

*Corresponding author: Sheng CHEN, Email: shechen@cityu.edu.hk.

Key words: Otilonium bromide, Colistin adjuvant, Membrane potential**,** Enterobacteriaceae, Drug repurposing

Supplementary Table 1. MICs of colistin tested in various colistin-resistant and susceptible clinical bacterial isolates with or without the presence of Ob.

Species	ID	Ob MIC (μg/ml)	Colistin MIC (μg/ml)		
		Ob	Colistin	Colistin+ 10ug/ml Ob	Colistin+ 20ug/ml Ob
Colistin-resistant Gram-negative strains					
E. coli	*mcr-1*-bearing *E. coli* J53	>64	8	1	0.25
E. coli	2016.2.5 1149-1	>64	8	4	1
E. coli	WZ3955	>64	8	4	1
E. coli	X2169	>64	16	4	2
E. coli	WZ2431	>64	8	2	0.5
E. coli	WZ3920	>64	4	2	≤0.25
E. coli	WZ2909	>64	16	8	1
E. coli	5	>64	8	4	≤0.25
E. coli	CX116	>64	2	≤0.25	≤0.25
E. coli	WZ3903	>64	4	≤0.25	≤0.25
E. coli	101	>64	8	4	1
E. coli	CX48	>64	8	4	1
E. coli	WZ3906	>64	8	4	1
E. coli	812	>64	16	4	1
E. coli	WZ3951	>64	16	8	2
E. coli	12.12.0487	>64	8	2	0.5
E. coli	34650	>64	8	2	0.5
E. coli	CX53	>64	8	4	2
E. coli	119	>64	16	4	2
A. baumannii	ABJZ-45	20	4	0.0625	-
A. baumannii	ABJZ-46	20	4	0.5	-
A. baumannii	ABJZ-50	20	4	0.25	-
A. baumannii	ABJZ-72	20	4	0.125	-
A. baumannii	ATCC17978	20	4	0.125	-
Colistin-susceptible Gram-negative strains					
S. Typhimurium	PY01	>64	2	0.125	0.03
E. coli	Bw25113	>64	2	0.03	≤0.015
P. aeruginosa	PAO1	>64	2	0.5	0.25
P. aeruginosa	PAERC 10.296	>64	2	0.0625	0.0625
P. aeruginosa	PAERC 11.2309	>64	2	0.5	2
P. aeruginosa	PAERC 11.236	>64	2	0.5	0.5
P. aeruginosa	PAERC 11.603	>64	2	0.5	0.25

P. aeruginosa	PAERC 12.1478	>64	2	0.5	0.125
P. aeruginosa	PAER 2011-3010	>64	2	1	0.25
P. aeruginosa	PAER 2011-3252	>64	2	0.5	0.25
P. aeruginosa	PAER 2012-1527	>64	2	2	0.5

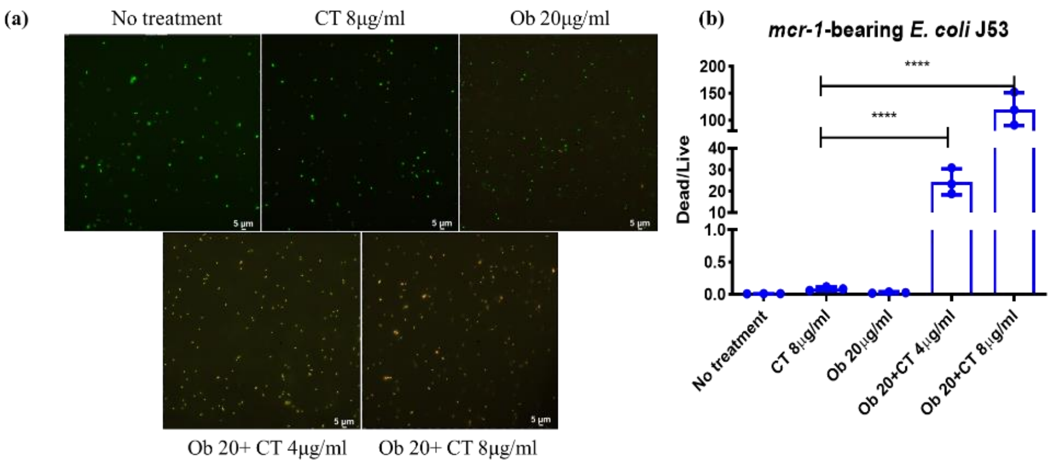

Supplementary Figure 1. LIVE/DEAD Cell Viability Assays depicting the efficacy of Ob and colistin combination therapy *in vitro*. (a) Microscopic observation of *mcr-1*-bearing *E. coli* stained with live and death dye was treated with colistin, Ob and different combinations of both. (b) Quantification of fluorescence signals of random views of 100 cells as presented in (a). Data are representative of three experiments performed in triplicate. Data are mean ± SEM for n=3 biologically independent experiment. *, p<0.05 , **, p<0.005 , *** p<0.0005 , **** p<0.00005 (independent *t*-test).

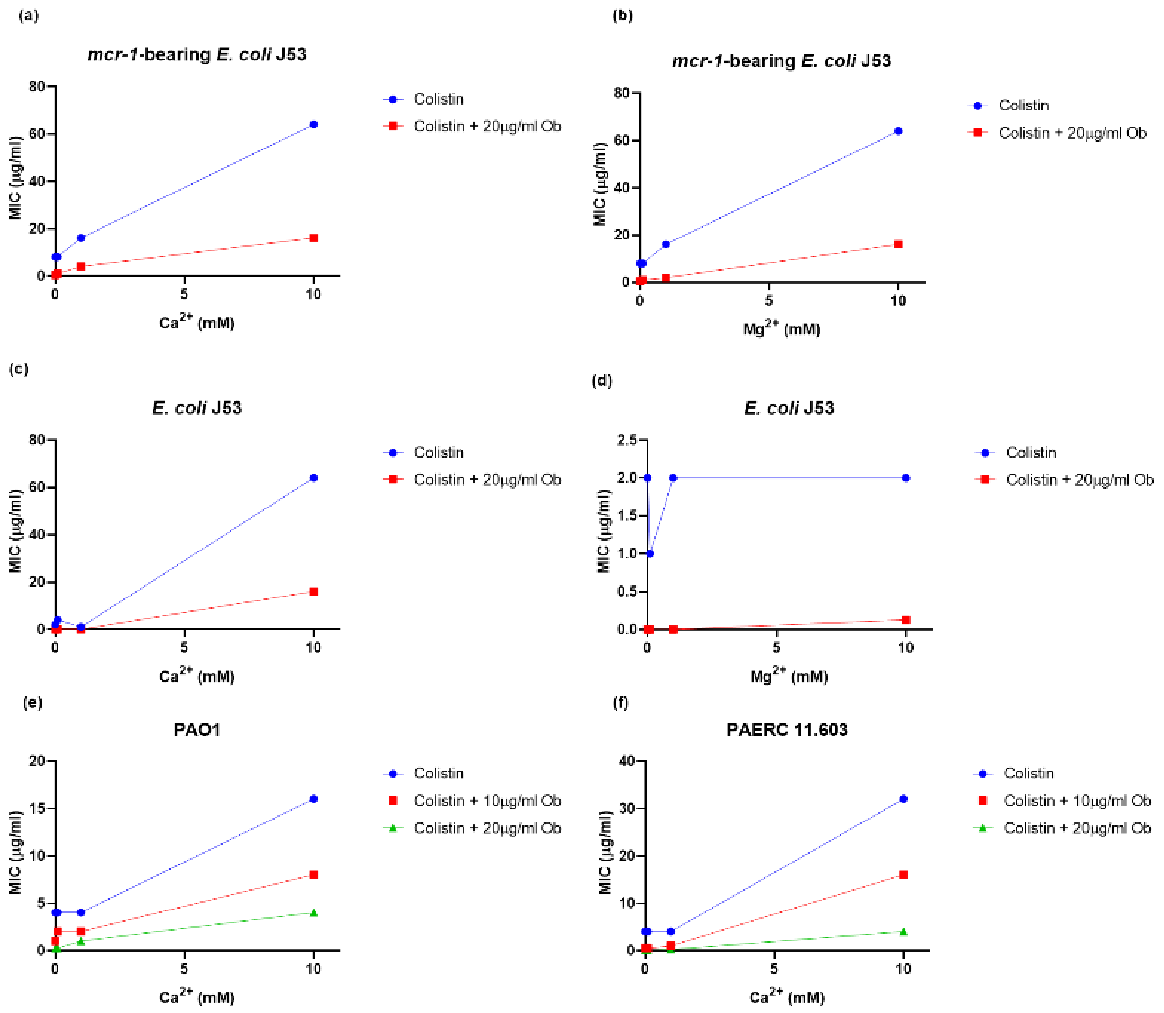

Supplementary Figure 2. The synergistic antibacterial effect of Ob and colistin can be suppressed by high concentrations of divalent cations. (a,b) *E. coli* J53 expressing MCR-1 was cultured in MHB and treated with colistin and Ob supplemented with different concentrations of Mg^{2+} and Ca^{2+} ions; (c,d) Colistin-susceptible *E. coli* J53 cultured in MHB was treated with colistin and Ob supplemented with different concentrations of Mg^{2+} and Ca^{2+} ions; Colistin MICs of *P. aeruginosa* PAO1 (e) and PAERC 11.603 (f) were determined upon supplementation with different concentrations of Ca^{2+} ions.

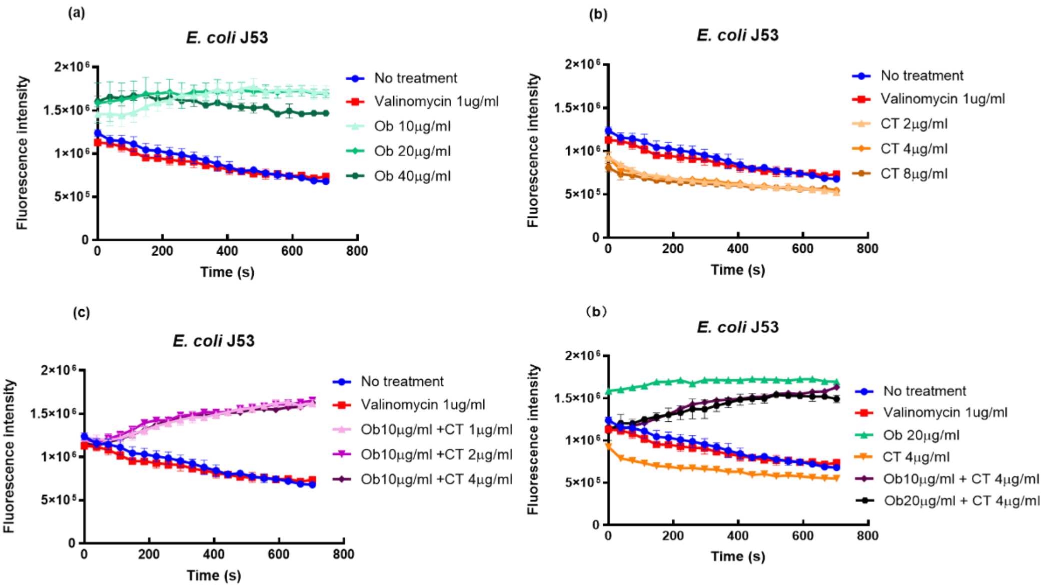

Supplementary Figure 3[1]. Dissipation of membrane potential in a colistin-susceptible *E. coli* J53 strain treated with Ob (a), colistin (b) and various combinations of the two drugs (c). (d) Comparison of effects of different compounds on the bacterial cell membrane potential of *mcr-1*-bearing *E. coli* J53.Valinomyxin, a K^+ ionophore, was used as the positive control. The assay was conducted in the presence of 100mM KCl. CT, colistin. Data are mean ± SEM for n=3 biologically independent experiment.
